# Diagnostic Accuracy of Ultrasound Elastography Versus Fine-Needle Aspiration Cytology in Predicting Benign and Malignant Thyroid Nodules in Solitary and Multinodular Thyroid Glands: A Comparative Analysis

**DOI:** 10.7759/cureus.72811

**Published:** 2024-11-01

**Authors:** Amanullah Khan, Muhammad Imran Siddiqui, Kamran Illahi Memon, Muhammad Imran Farid, Rabia Jaffar, Syed Naseer Ahmed, Zain Nayyer, Niamat Ullah, Muhammad Ahsan, Abdullah Ghumman

**Affiliations:** 1 Imaging, Cleveland Clinic, Abu Dhabi, ARE; 2 Clinical Imaging, Sheikh Shakhbout Medical City, Abu Dhabi, ARE; 3 Electrical and Computer Engineering, Air University, Islamabad, PAK; 4 Internal Medicine, Liaquat University of Medical and Health Sciences, Jamshoro, PAK; 5 Radiology, Sheikh Khalifa Bin Zayed Hospital, Quetta, PAK; 6 Diagnostic Radiology, Combined Military Hospital, Gujranwala, PAK; 7 General Surgery, Lady Reading Hospital Medical Teaching Institute, Peshawar, PAK; 8 Internal Medicine, Jinnah Postgraduate Medical Centre, Karachi, PAK; 9 Medicine and Surgery, King Edward Medical University, Lahore, PAK

**Keywords:** benign, diagnostic accuracy, fine-needle aspiration cytology, fnac, malignant, thyroid nodules, ultrasound elastography

## Abstract

Background

The diagnosis of thyroid nodules requires accurate techniques; fine-needle aspiration cytology (FNAC) is the gold standard, while ultrasonography elastography has potential but is not well supported by data, particularly when it comes to differentiating benign from malignant nodules in single and multiple noduled glands.

Objective

This study's main goal was to assess the diagnostic accuracy of ultrasound elastography in predicting benign versus malignant thyroid nodules, both in solitary and multinodular thyroid glands, compared with FNAC.

Methodology

This prospective observational study evaluated thyroid nodules using ultrasound elastography and FNAC. Patients who were 18 years of age or older and had visible thyroid nodules met the inclusion criteria; those who had undergone thyroid surgery in the past, had cancer, or refused both tests were excluded. Data on demographics, clinical conditions, and imaging were gathered from 360 enrolled patients. Statistical analysis included calculating sensitivity, specificity, and diagnostic accuracy of ultrasound elastography using FNAC as a reference, alongside receiver operating characteristic curve analysis to determine the optimal cutoff for benign versus malignant nodules.

Results

There were 360 individuals in the trial, 250 of whom had benign thyroid nodules and 110 of whom had malignant ones. When compared to ultrasound elastography, FNAC showed somewhat better sensitivity (92.00%) and specificity (85.33%) for benign nodules. On the other hand, for malignant nodules, FNAC showed better specificity (80.95%) and sensitivity (91.82%) than ultrasonic elastography. In all age categories, FNAC consistently performed better than ultrasound elastography. The total accuracy obtained by ultrasound elastography was 81.94%, but the accuracy obtained by FNAC was higher at 85.47%. Ultrasound elastography's ideal cutoff value was found to be 4.2, with a sensitivity of 87.25% and a specificity of 78.40%.

Conclusion

Ultrasound elastography shows significant promise as a non-invasive, real-time complementary tool to FNAC for diagnosing thyroid nodules.

## Introduction

Ultrasound imaging investigations have shown that thyroid nodules are a frequent clinical entity seen in regular medical practice, with an estimated incidence of up to 68% in some populations [1,2]. While most thyroid nodules are benign, a sizable fraction are cancerous, requiring precise diagnostic instruments for prompt treatment and care [3,4]. For evaluating thyroid nodules, fine-needle aspiration cytology (FNAC) has long been considered the gold standard because it offers important details on the cytological features of the nodules [5]. FNAC does have several drawbacks, however, such as the possibility of ambiguous findings and the need for additional processes in certain circumstances [6].

Ultrasound elastography has been a viable supplementary technique for the assessment of thyroid nodules in recent times. It provides useful information on the tissue elasticity of these lesions and may help distinguish between benign and malignant ones [7,8]. Through the use of this non-invasive imaging technology, doctors may further stratify nodules according to their risk of malignancy, since malignant nodules often demonstrate higher stiffness than their benign counterparts [9].

There is a lack of high-quality evidence regarding the diagnostic accuracy of ultrasound elastography, even with the growing body of literature exploring its usefulness in thyroid nodule evaluation. This is especially true when it comes to differentiating between benign and malignant nodules in both solitary and multinodular thyroid glands [10,11]. Studies that have already been conducted have produced contradictory findings; some have shown excellent sensitivity and specificity, while others have shown more moderate performance characteristics [12].

To maximize the clinical value of ultrasound elastography and improve its place in the thyroid nodule diagnosis algorithm, this research gap must be filled. Explaining the diagnostic accuracy of ultrasound elastography in differentiating between benign and malignant nodules, especially when compared to FNAC, will help doctors make better judgments about patient care and treatment plans.

Research objective

This study's main goal was to assess the diagnostic accuracy of ultrasound elastography in predicting benign versus malignant thyroid nodules, both in solitary and multinodular thyroid glands, when compared with FNAC.

## Materials and methods

Ethical approval

The study was conducted after obtaining ethical approval from the Abu Dhabi Health Research and Technology Ethics Committee (approval number: DOH/CVDC/2022/213) and finalizing the protocol before data collection began. All patients gave their informed permission before being included in the research, and patient anonymity was conscientiously maintained the whole time the study was conducted.

Study design and settings

This prospective observational study was conducted in Cleveland Clinic Abu Dhabi, Sheikh Shakhbout Medical City Abu Dhabi, Sheikh Khalifa Bin Zayed Hospital Quetta, Combined Military Hospital Gujranwala, and Medical Teaching Institute Lady Reading Hospital Peshawar from January to December 2023. Modern diagnostic imaging and pathology facilities at these institutes make them the perfect place for a thorough assessment of thyroid nodules.

Inclusion and exclusion criteria

Participants in the research had to be at least 18 years old and present to the outpatient department with thyroid nodules diagnosed either incidentally or clinically during imaging investigations. This study specifically included patients with thyroid nodules found incidentally during ultrasound examinations of the neck, provided they were not clinically palpable at the time of examination. Additionally, patients presenting with cervical lymphadenopathy who were subsequently found to have small malignant thyroid masses were also included. The research excluded patients who had undergone thyroid surgery or radiation in the past, had known thyroid cancer, or were unable to undertake both ultrasound elastography and FNAC.

Sample size

The research included 360 consecutive patients who satisfied the inclusion criteria. The expected frequency of thyroid nodules in the study population and the required degree of precision for calculating the precision of diagnosis of ultrasound elastography associated with FNAC were taken into consideration while determining this sample size.

Data collection

Demographic and clinical data, including age, gender, presenting symptoms, and relevant medical history, were recorded for each patient. Every patient who was included had thyroid ultrasound elastography and then FNAC, which were carried out in accordance with established procedures by skilled radiologists and pathologists, respectively. The elasticity score was used to subjectively evaluate the ultrasonic elastography pictures, and the FNAC findings were categorized as benign, malignant, or undetermined.

Statistical analysis

Statistical analysis was performed using SPSS Statistics version 27 (IBM Corp. Released 2020. IBM SPSS Statistics for Windows, Version 27.0. Armonk, NY: IBM Corp). The evaluation of ultrasonic elastography's specificity, sensitivity, positive predictive value (PPV), negative predictive value (NPV), and diagnostic efficacy in differentiating benign from malignant thyroid nodules was conducted using FNAC as the gold standard. The receiver operating characteristic (ROC) curve was examined in order to determine the optimal cutoff value for differentiating between benign and malignant nodules.

## Results

Out of 360 patients, 250 had benign nodules and 110 had malignant nodules. Table 1 gives a detailed summary of the features of both benign and malignant thyroid nodules. The age distribution shows that patients with benign nodules are most likely to be between the ages of 30 and 50, making up 151 patients (60.40%). Patients over 50 make up the second largest group, with 71 patients (28.40%), and patients under 30 make up the smallest group, with 28 patients (11.20%). On the other hand, among patients who have malignant nodules, the majority fall into the 30- to 50-year-old age group (57, 51.82%), followed by patients over 50 (44, 40.00%), and patients under 30 (9, 8.18%) have the lowest percentage. Patients with benign nodules had an average age of 45.76 years (SD ± 12.34), whereas patients with malignant nodules had an average age of 50.82 years (SD ± 9.67). In both the benign (188, 75.20%) and malignant (67, 60.91%) nodule categories, the majority of patients are female. Palpable nodules are the predominant symptom found in 189 individuals (75.60%) with benign cases and 83 patients (75.45%) with malignant cases. Although they are less common, other symptoms including hoarseness, neck discomfort, dysphagia, and inadvertent detection are also reported. Comparable rates of hypertension, diabetes, thyroid disorders, and smoking history are found in the medical histories of the two groups; percentages for benign nodules range from 34 patients (13.60%) to 94 patients (37.60%), while those for malignant nodules range from 15 patients (13.64%) to 43 patients (39.09%).

**Table 1:** Characteristics of benign versus malignant thyroid nodules

Characteristic		Benign nodules (n=250)		Malignant nodules (n=110)	
		n	%	n	%
Age group	<30 years	28	11.20	9	8.18
	30-50 years	151	60.40	57	51.82
	>50 years	71	28.40	44	40.00
Age (years)	Mean ± SD	45.76 ± 12.34		50.82 ± 9.67	
Gender	Male	62	24.80	43	39.09
	Female	188	75.20	67	60.91
Symptoms	Palpable	189	75.60	83	75.45
	Incidental	46	18.40	18	16.36
	Neck pain	38	15.20	29	26.36
	Dysphagia	23	9.20	21	19.09
	Hoarseness	18	7.20	13	11.82
Medical history	Hypertension	51	20.40	21	19.09
	Diabetes	34	13.60	15	13.64
	Thyroid disorder	71	28.40	31	28.18
	Smoking	94	37.60	43	39.09

Table 2 presents the distribution of 360 thyroid nodules among solitary and multinodular glands. In glands with multiple nodules, 102 (28.33%) were benign and 41 (11.39%) were malignant. Of the solitary nodules, 148 (41.11%) were benign and 69 (19.17%) were malignant. Additionally, the distribution of nodules by site reveals that 44 (12.22%) of the solitary nodules were located in both lobes, 71 (19.72%) in the left lobe, and 102 (28.33%) in the right lobe. Comparatively, among multinodular glands, 67 (18.61%) nodules were found in the right lobe, 52 (14.44%) in the left lobe, and 24 (6.67%) in both lobes.

**Table 2:** Distribution of thyroid nodules and comparison of sensitivity and specificity by nodule type FNAC: fine-needle aspiration cytology, n: number of participants

Nodule type		n	%	Ultrasound elastography		FNAC	
				Sensitivity (n)	Specificity (%)	Sensitivity (n)	Specificity (%)
						Benign	Solitary	148	41.11	-	-	-	-
Multinodular	102	28.33	225	89.60	229		92.00
Malignant	Solitary	69	19.17	-	-	-	-
	Multinodular	41	11.39	92	83.64	101	91.82
Location	Right lobe	102	28.33	96	76.19	89	80.95
	Left lobe	71	19.72	-	-	-	-
	Both lobes	44	12.22	-	-	-	-

The sensitivity and specificity of FNAC and ultrasound elastography for detecting benign and malignant nodules are also outlined in Table 2. Ultrasound elastography demonstrated a sensitivity of 89.60% (n=225) and specificity of 79.33% among the 250 benign nodules, whereas FNAC showed slightly higher sensitivity and specificity at 92.00% (n=229) and 85.33%, respectively. For the 110 malignant nodules, ultrasound elastography had a sensitivity of 83.64% (n=92) and specificity of 76.19% (n=96), while FNAC showed higher sensitivity at 91.82% (n=101) and specificity at 80.95% (n=89). These results suggest that while ultrasound elastography is a valuable tool, FNAC generally provides superior diagnostic accuracy for both benign and malignant thyroid nodules.

**Figure 1:**
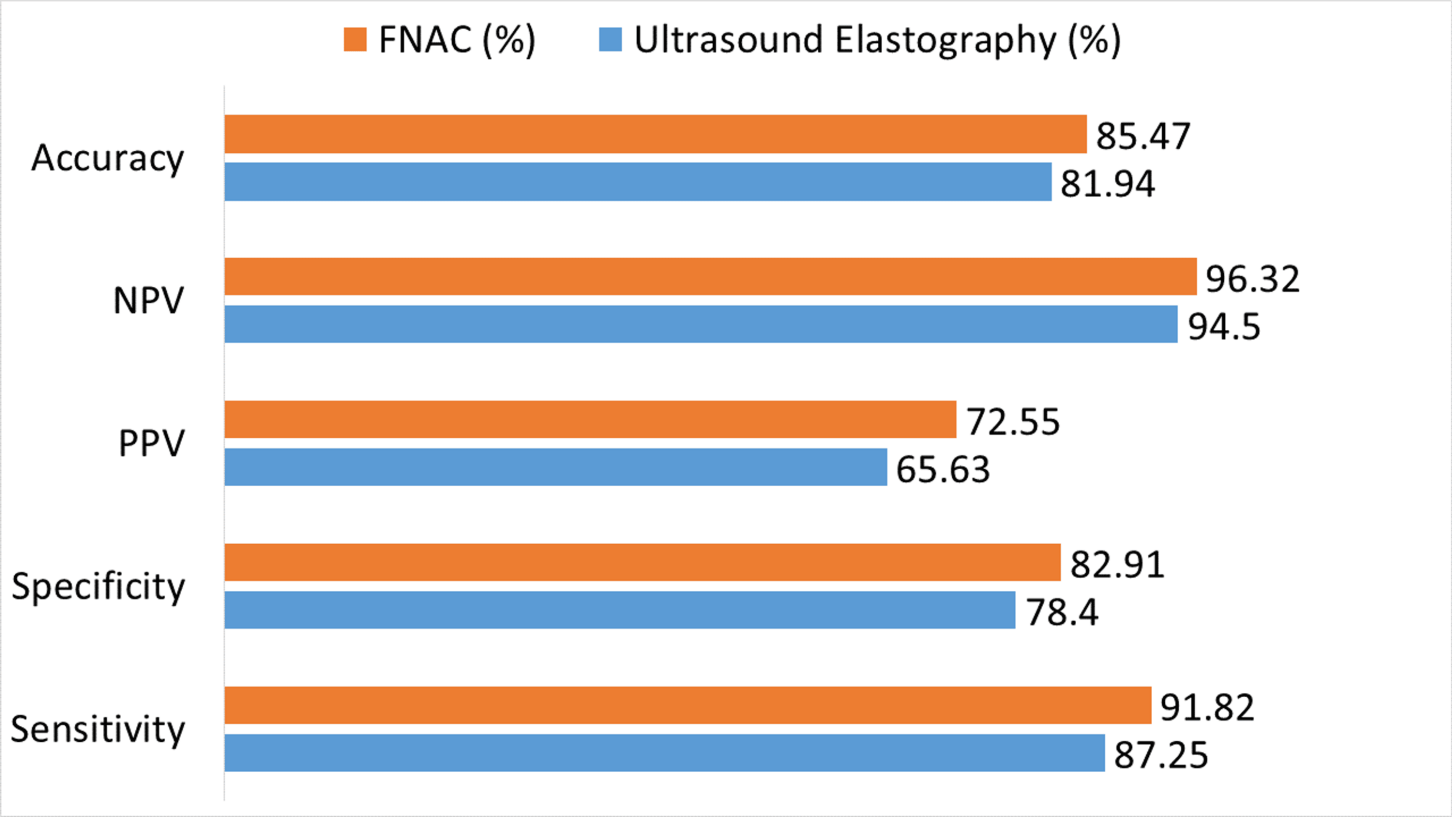
Diagnostic accuracy of ultrasound elastography versus FNAC PPV: positive predictive value, NPV: negative predictive value, FNAC: fine-needle aspiration cytology

Figure 1 contrasts the ability of FNAC and ultrasound elastography to diagnose benign versus malignant thyroid nodules. The results of ultrasound elastography show that it has an 81.94% overall accuracy, with a sensitivity of 87.25%, specificity of 78.40%, PPV of 65.63%, and NPV of 94.50%. On the other hand, FNAC has a greater accuracy of 85.47%, PPV of 72.55%, NPV of 96.32%, specificity of 82.91%, and sensitivity of 91.82%.

The diagnostic accuracy of FNAC and ultrasound elastography is shown in Table 3 according to age group. There were a total of 250 patients in the <30 age group (n=28), 151 patients in the 30-50 age group (n=151), and 71 patients in the >50 age group (n=75). Within the sub-30 age range, FNAC has somewhat greater specificity (96.43%) and sensitivity (89.29%) than ultrasonic elastography (78.57% and 75.00%, respectively). Ultrasound elastography has a sensitivity of 85.43% and a specificity of 78.15% in patients aged 30 to 50, whereas FNAC shows a greater sensitivity of 91.86% and a similar specificity of 84.78%. Ultrasound elastography has a sensitivity of 81.69% and a specificity of 74.65% for individuals over 50, but FNAC has a greater sensitivity of 88.73% and a comparable specificity of 80.28%.

**Table 3:** Diagnostic accuracy of ultrasound elastography and FNAC by age group FNAC: fine-needle aspiration cytology

Age group	Ultrasound elastography				FNAC			
	Sensitivity		Specificity		Sensitivity		Specificity	
	n	%	n	%	n	%	n	%
<30 (n=28)	22	78.57	21	75.00	25	89.29	27	96.43
30-50 (n=151)	129	85.43	118	78.15	139	91.86	128	84.78
>50 (n=71)	58	81.69	53	74.65	63	88.73	57	80.28

The ideal cutoff value for ultrasonic elastography is shown in Table 4, where 4.2 is the value. Ultrasound elastography has 87.25% sensitivity and 78.40% specificity at this level. These results show how well ultrasound elastography predicts benign from malignant thyroid nodules; at the given cutoff value, there is a high sensitivity for properly detecting malignant nodules and a moderate specificity for correctly identifying benign nodules.

**Table 4:** Optimal cutoff value for ultrasound elastography

Parameter	Cutoff value	Sensitivity (%)	Specificity (%)
Elastography score	4.2	87.25	78.40

**Figure 2:**
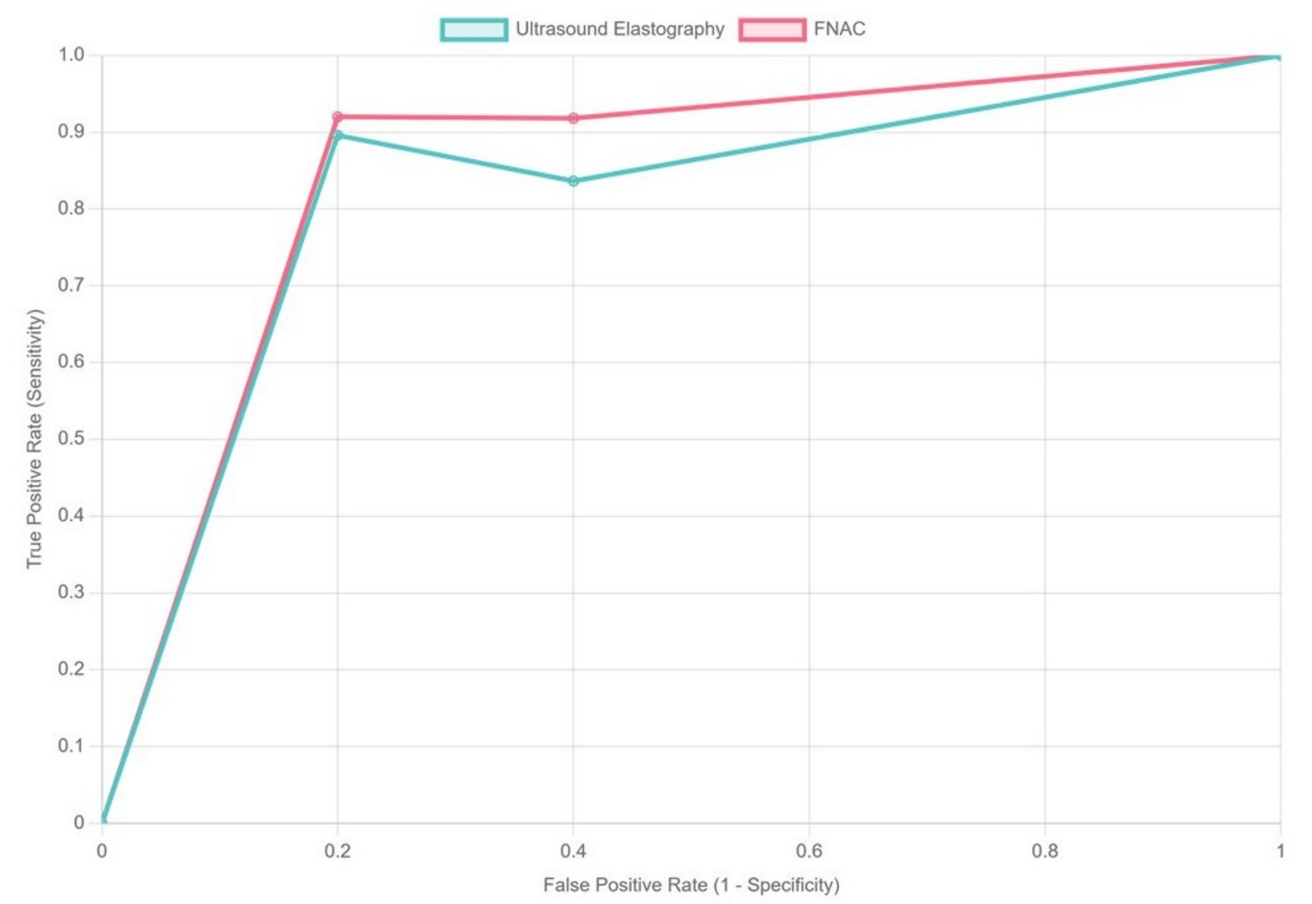
ROC curve for ultrasound elastography and FNAC ROC: receiver operating characteristic, FNAC: fine-needle aspiration cytology

The ROC curve in Figure 2 provides a visual representation of the diagnostic performance of ultrasound elastography and FNAC in distinguishing between benign and malignant thyroid nodules. The area under the ROC curve (AUC) quantifies the overall accuracy of these diagnostic methods, with values closer to 1 indicating better performance. In this case, ultrasound elastography demonstrated an AUC of approximately 0.84, while FNAC had a slightly higher AUC of around 0.88. These results suggest that both diagnostic techniques are effective, but FNAC provides superior accuracy in differentiating between benign and malignant nodules. The cutoff value of 4.2 for ultrasound elastography, which exhibited a sensitivity of 87.25% and a specificity of 78.40%, indicates that this method can effectively identify malignant cases while maintaining a reasonable rate of false positives. This balance is crucial in clinical practice, as it impacts patient management and treatment decisions.

Moreover, the ROC curve highlights the trade-off between sensitivity and specificity across different thresholds. For instance, adjusting the cutoff value can enhance sensitivity at the cost of specificity or vice versa. Clinicians may opt for a lower cutoff to maximize sensitivity when they prioritize identifying malignant nodules, which is essential for timely intervention. A higher cutoff may be preferred to increase specificity and minimize unnecessary procedures in patients with benign nodules. Overall, the ROC analysis provides valuable insights into the effectiveness of these diagnostic modalities, guiding clinicians in selecting the most appropriate tests based on patient-specific factors, age group, and symptomatology. Understanding the implications of these results can aid in optimizing diagnostic strategies and improving patient outcomes in the management of thyroid nodules.

Table 5 shows that the ultrasound imaging reveals a right hyperechoic, well-defined nodule measuring 2.5 x 1.8 cm, with evidence of extra-thyroid extension and no vascularity on color Doppler imaging. In contrast, the left thyroid appears normal, measuring 1.6 x 1.4 cm and showing normal blood flow on color Doppler imaging. The isthmus is also normal, with a thickness of 0.3 cm. The impression is that the right thyroid nodule is classified as Thyroid Imaging Reporting and Data Systems (TIRADS) 5.

**Table 5:** Summary of nodule characteristics, thyroid lobe sizes, vascularity, and TIRADS classification for both right and left thyroid lobes, as well as the isthmus TIRADS: Thyroid Imaging Reporting and Data Systems, N/A: not applicable

Clinical findings	Right thyroid lobe	Left thyroid lobe	Isthmus
Nodule presence	Hyperechoic, well-defined nodule, and few small nodules	No nodule and multiple nodules	N/A
Nodule size	Largest: 2.5 x 1.8 cm (hyperechoic); 0.8 x 0.6 cm (hypoechoic to isoechoic)	Largest: 1.3 x 0.8 cm (heterogenous solid cystic with calcification)	N/A
Extra-thyroid extension	Present	N/A	N/A
Vascularity (color Doppler)	No vascularity	Normal blood flow	N/A
Thyroid gland appearance	Enlarged, heterogeneous	Enlarged, heterogeneous	Normal
Thyroid lobe size	1.9 x 1.8 cm	2 x 2 cm	Thickness: 0.8 cm
TIRADS classification	TIRADS 5	N/A	N/A
Impression	Suspicious (malignant features)	Normal	Normal

In addition, an enlarged and heterogeneous thyroid gland was observed. The right thyroid lobe measures 1.9 x 1.8 cm and contains a few small nodules, the largest being 0.8 x 0.6 cm, which is hypoechoic to isoechoic. The left thyroid lobe, which measures 2 x 2 cm, contains multiple nodules, including a heterogeneous, well-defined solid cystic nodule with internal calcification measuring 1.3 x 0.8 cm. The isthmus appears normal, with a thickness of 0.8 cm.

The distribution of thyroid nodules by size is shown in Table 6, along with the percentage (%) and absolute count (n) of benign and malignant cases. Of the 250 benign nodules, 119 (47.60%) varied in size from 1-2 cm, 58 (23.20%) were bigger than 2 cm, and 73 (29.20%) were smaller than 1 cm. On the other hand, of the 110 malignant nodules, 27 (24.55%) had a size of less than 1 cm, 58 (52.73%) had a size of between 1-2 cm, and 25 (22.73%) had a size more than 2 cm.

**Table 6:** Distribution of thyroid nodules by size

Nodule size	Benign nodules (n=250)		Malignant nodules (n=110)	
	n	%	n	%
<1 cm	73	29.20	27	24.55
1-2 cm	119	47.60	58	52.73
>2 cm	58	23.20	25	22.73

**Figure 3:**
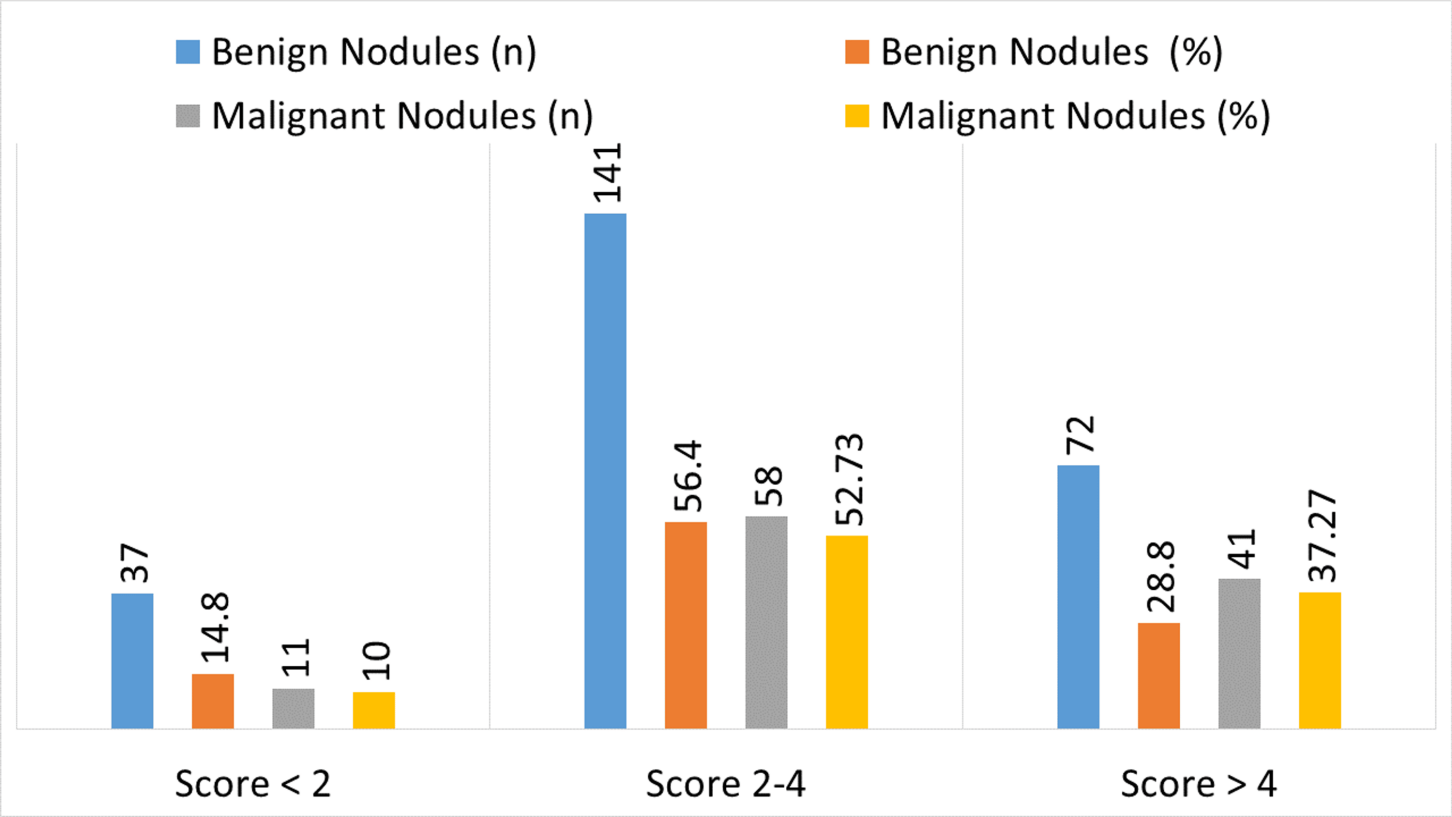
Distribution of ultrasound elastography scores

The distribution of ultrasound elastography scores for thyroid nodules, both benign and malignant, is shown in Figure 3. Of the benign nodules, 141 (56.40%) had elastography scores between 2 and 4, 72 (28.80%) had scores above 4, and 37 (14.80%) had scores below 2. By comparison, of the malignant nodules, 41 (37.27%) had scores over 4, 58 (52.73%) ranged between 2 and 4, and 11 (10.00%) had values below 2.

## Discussion

This research compared the diagnostic accuracy of ultrasonic elastography with FNAC in identifying benign versus malignant thyroid nodules. The findings show that, while there are differences in sensitivity and specificity across age groups, ultrasonic elastography has promising performance. Ultrasound elastography showed that of the 360 patients examined, benign nodules had a sensitivity of 89.60% and a specificity of 79.33%, whereas malignant nodules showed a sensitivity of 83.64% and a specificity of 76.19%. These results are consistent with other studies showing the potential of ultrasound elastography as a useful supplementary instrument in thyroid nodule assessment [13-15].

When compared to FNAC, the results showed that FNAC had somewhat better sensitivity and specificity values, indicating that it is preferable in certain areas of thyroid nodule diagnosis. For benign nodules, FNAC had a sensitivity of 92.00% and a specificity of 85.33%, whereas for malignant nodules, the corresponding values were 91.82% and 80.95%, respectively. Although FNAC is still the gold standard, there are benefits to ultrasonic elastography such as non-invasiveness and real-time evaluation that may increase its therapeutic value, especially when FNAC findings are unclear or repeat procedures are required, as also stated in other studies [16,17].

The assessment of ultrasonic elastography performance in various age groups produced several thought-provoking findings. FNAC showed greater specificity (96.43%) and sensitivity (89.29%) in the age group under 30 than ultrasonic elastography (78.57% sensitivity and 75.00% specificity). When compared to FNAC (91.86%) in the 30- to 50-year-old age range, ultrasound elastography had similar sensitivity (85.43%) but significantly lower specificity (78.15% for ultrasound elastography against 84.78% for FNAC). Similarly, FNAC showed similar specificity (80.28%) and better sensitivity (88.73%) in the >50 age group compared to ultrasonic elastography (81.69% sensitivity and 74.65% specificity). These results are similar to other studies and highlight the significance of taking age-related differences in diagnostic accuracy into account when interpreting the results of ultrasonography elastography [18,19].

The diagnostic efficacy of ultrasonic elastography is further enhanced by the ideal cutoff value of 4.2. Ultrasound elastography showed promise in differentiating between benign and malignant thyroid nodules at this cutoff value, with a sensitivity of 87.25% and specificity of 78.40%. When analyzing ultrasound elastography results and making choices about patient care, this cutoff value gives doctors a useful point of reference [20,21].

Limitations

All things considered, FNAC remains the gold standard for evaluating thyroid nodules, but ultrasound elastography shows potential as a supplemental method, particularly when FNAC findings are unclear or require further investigation. However, one limitation of this study is its observational design, which may introduce selection bias, especially since it was conducted across multiple centers with varying expertise in imaging and pathology. Additionally, the exclusion of patients with prior thyroid surgery, radiation, or known thyroid cancer limits the generalizability of the findings to a broader patient population. The reliance on subjective elastography scoring and FNAC interpretations might result in variability, potentially affecting diagnostic accuracy. Moreover, the relatively short duration of the study (one year) limits the assessment of long-term outcomes related to the thyroid nodules examined. Further research is needed to confirm these findings and explore potential synergies between FNAC and ultrasound elastography in enhancing diagnostic precision and guiding clinical decision-making in thyroid nodule management. This study provides a foundational step in understanding the complementary role of ultrasound elastography in thyroid nodule evaluation.

## Conclusions

Ultrasound elastography shows significant potential as a non-invasive, real-time adjunct to FNAC for diagnosing thyroid nodules. The advantages of ultrasonic elastography include its non-invasiveness and real-time evaluation capabilities, even if FNAC showed somewhat better sensitivity and specificity. According to this research, ultrasound elastography has an overall diagnostic accuracy of 81.94%. Its usefulness in differentiating between benign and malignant nodules is enhanced by the use of suitable cutoff values. These data demonstrate the usefulness of ultrasonic elastography, especially when FNAC results are unclear or need numerous treatments.
